# Supplementary material for: Neural silences can be localized rapidly using noninvasive scalp EEG
Source: Commun Biol. 2021 Mar 30;4:429. doi: 10.1038/s42003-021-01768-0 (PMC8010113; doi:10.1038/s42003-021-01768-0)
Supplement: Supplementary file 2 — Supplementary Information [file 42003_2021_1768_MOESM2_ESM.pdf]

Supplementary materials for
"Neural silences can be localized rapidly using noninvasive scalp EEG"

Alireza Chamanzar[1,2*], **Marlene Behrmann**[2,3], **and Pulkit Grover**[1,2*]
[1]Electrical and Computer Engineering Department, Carnegie Mellon University, Pittsburgh, PA, USA.
[2]Neuroscience Institute, Carnegie Mellon University, Pittsburgh, PA, USA.
[3]Psychology Department, Carnegie Mellon University, Pittsburgh, PA, USA.
[*]correspondence to: {pgrover, achamanz}@andrew.cmu.edu.

This file includes:

- **Supplementary Note A-H**
- **Supplementary Fig. 1-11**
- **Supplementary Table I and II**
- **Supplementary References**

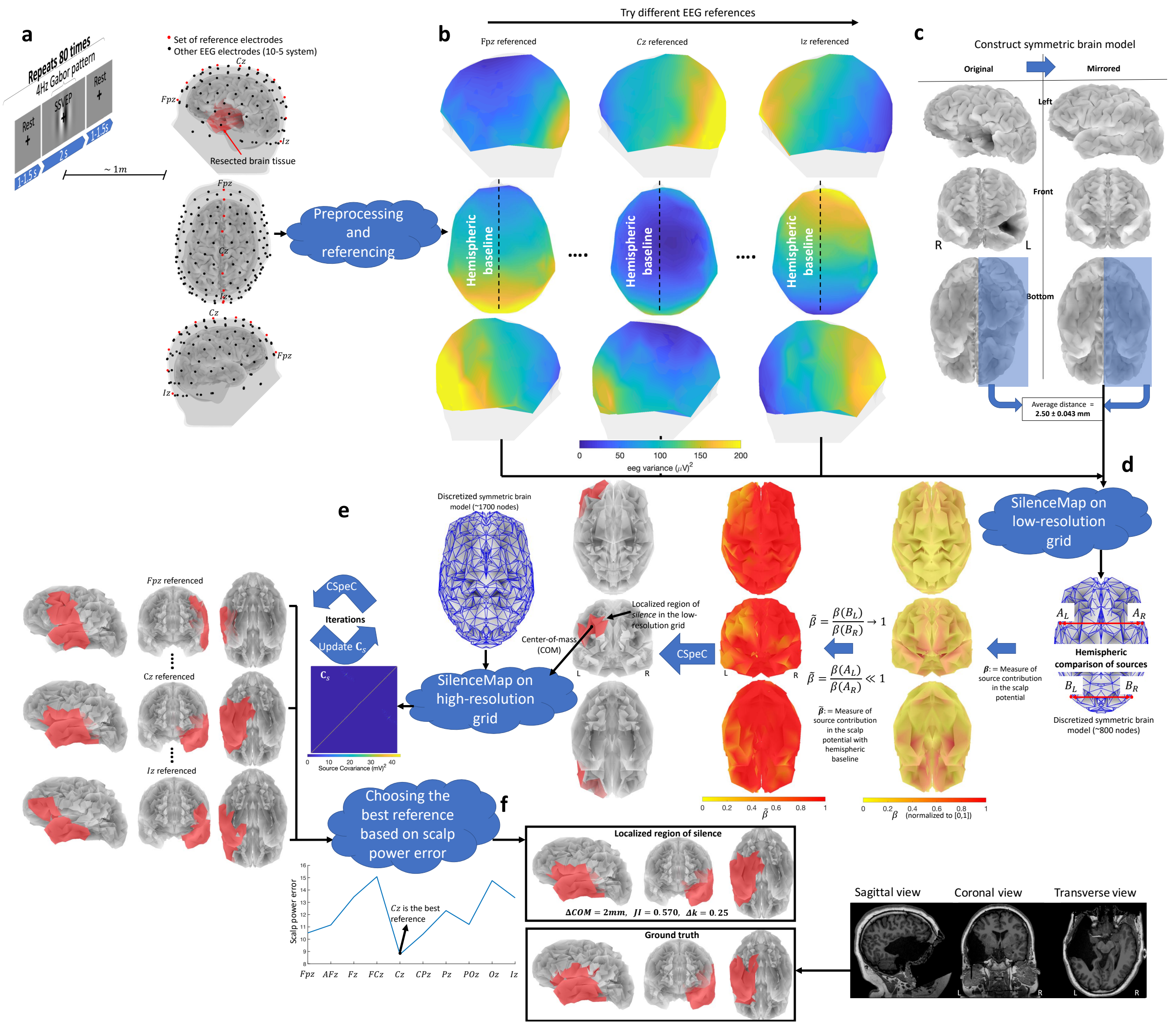

Supplementary Fig. 1. **SilenceMap with baseline algorithm overview based on patient SN's Rest dataset:** a) The EEG recording protocol and the locations of scalp electrodes. One of 10 reference electrodes (shown in red) is chosen along the longitudinal fissure for rereferencing against. b) Average power of scalp potentials for different choices of reference electrodes. c) Symmetric brain model of a patient (SN) with left temporal hematoma. d) Steps of the SilenceMap algorithm in a low-resolution source grid. A measure of the contribution of brain sources in the recorded scalp signals ($\tilde{\beta}$) is calculated relative to a hemispheric baseline. In the brain colormap, yellow indicates no contribution. A contiguous region of silence is localized based on a convex spectral clustering (CSpeC) framework in the low-resolution grid. e) Steps of the SilenceMap algorithm in a high-resolution source grid. The source covariance matrix ($\mathbf{C}_s$) is estimated through an iterative method, and the region of silence is localized using the CSpeC framework. f) Choosing the best reference electrode to reference against (Cz in this example), which results in minimum scalp power mismatch (ΔPow). The localized region of silence for this patient (SN) has 2mm COM distance (ΔCOM) from the original region, with more than 57% overlap (JI = 0.570), and it is 25% larger ($\Delta k = 0.25$).

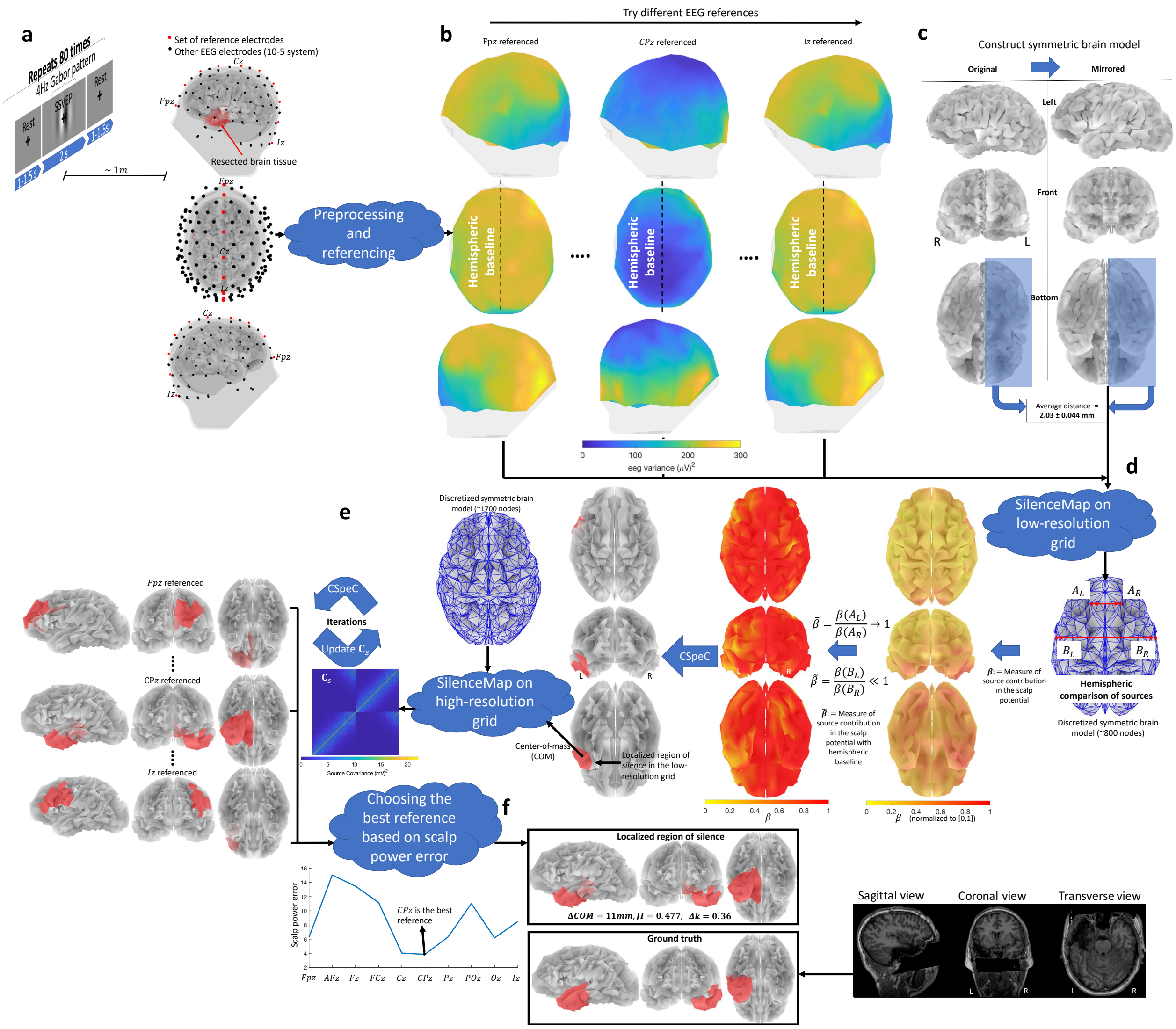

Supplementary Fig. 2. **SilenceMap with baseline algorithm overview based on patient OT's Rest dataset:** a) The EEG recording protocol and the locations of scalp electrodes. One of 10 reference electrodes (shown in red) is chosen along the longitudinal fissure for rereferencing against. b) Average power of scalp potentials for different choices of reference electrodes. c) Symmetric brain model of a patient (OT) with left temporal resection. d) Steps of the SilenceMap algorithm in a low-resolution source grid. A measure of the contribution of brain sources in the recorded scalp signals ($\tilde{\beta}$) is calculated relative to a hemispheric baseline. In the brain colormap, yellow indicates no contribution. A contiguous region of silence is localized based on a convex spectral clustering (CSpeC) framework in the low-resolution grid. e) Steps of the SilenceMap algorithm in a high-resolution source grid. The source covariance matrix ($\mathbf{C}_s$) is estimated through an iterative method, and the region of silence is localized using the CSpeC framework. f) Choosing the best reference electrode to reference against (CPz in this example), which results in minimum scalp power mismatch (ΔPow). The localized region of silence for this patient (OT) has 11mm COM distance (ΔCOM) from the original region, with more than 47% overlap (JI = 0.477), and it is 36% smaller ($\Delta k = 0.36$).

Supplementary Note A. The list of all parameters and their values used in the SilenceMap algorithm and modified source localization algorithms

The values of all parameters we have used to implement and test the SilenceMap algorithm, with and without baseline, as well as the modified source localization algorithms are summarized in Supplementary Table I. All code and datasets are freely available online in [1, 2].

Supplementary Table I. Parameters used for implementation of the SilenceMap, modified MNE, MUSIC, and sLORETA algorithm.

Name	Value
δ (Convergence parameter in SilenceMap)	1cm
$\hat{k}$ search grid (SilenceMap)	$[2, 7, 12, \cdots, 100]$
$\lambda^{\star}$ search grid (SilenceMap without baseline)	$[1, 3.2, 10, \cdots, 100] \times \sum_{q=1}^{p} \beta_q$
$\lambda^{\star}$ search grid (SilenceMap with baseline)	$[1, 3.2, 10, \cdots, 100] \times \sum_{q=1}^{p} \tilde{\beta}_q$
R (Maximum number of iterations in SilenceMap)	100
ϕ in equation (33) in our paper (SilenceMap)	90
z^{gap} (SilenceMap with baseline)	1cm
λ search grid (modified sLORETA and MNE)	$[0.01, 0.012, 0.016, \cdots, 100]$
k_0 (modified MNE and sLORETA)	$\lfloor \frac{p}{10} \rfloor$
ρ (modified MUSIC)	99

Supplementary Note B. Discussion on the performance of standard neuropsychological tests in silence localization

Neuropsychological testing might suffice for determining where the lesion is in many cases. Of course, the sensitivity of these measures is somewhat coarse and localization is often at the level of the lobe with some indication of whether the lesion is in the left or right hemisphere (e.g. if performance is poor on the Wisconsin Card Sorting Test or on Verbal/Visual fluency generation tasks, we might conclude that the lesion is in left or right frontal cortex or if performance is poor on list recall, we might conclude that the lesion is in temporal cortex and perhaps anterior and medial). Note that these measures do not permit characterization of the site or size of the lesion with any precision, and this contrasts directly with the localization achieved by SilenceMap algorithm. But perhaps even more relevant here is that, in the 3 cases presented here of children/adolescents with resection (see Table II in "Results"), there is rather minimal, if any, effect of the resection on behavioral performance, indicative of substantial plasticity in the children's brain. We do not have detailed behavioral data for SN, but we do know that he is at school at the grade level commensurate with his age. As shown in Supplementary Table II, for the other two patients, performance on neuropsychological tests post-surgically is high with only an occasional measure that is relatively low (e.g. 34th percentile for WISC in UD but post-surgical IQ is 118 (full scale), 123 (verbal), 108 (performance). We present scores of various neuropsychological measures for the patients (OT and UD) and show that, dramatically, notwithstanding the large resection, performance is very good [3, 4]. The use of a silence localization algorithm would therefore, provide an important source of lesion site and size in postsurgical cases.

Supplementary Table II. Pediatric patients' neuropsychological evaluation test performance pre- and post-surgery [4]

Patient	Hemisphere	Detailed IQ measures	Vision or visual motor integration	Memory learning	Executive function	Academic skills/performance
OT	left	***Pre-surgery:***<br>**WASI:** 122 (full scale),<br>125 (verbal), 114 (performance)<br>***Post-surgery:***<br>**WASI:** 127 (full scale)	**Grooved pegboard:**<br>average<br>(dominant hand)	**CVLT-C:**<br>high average<br>**WRAML-2:**<br>high average	**DKEFS:**<br>superior	**WJ III ACH:**<br>above age and grade expectancy
UD	right	***Pre-surgery:***<br>**WASI:** 116 (full scale)<br>135 (verbal), 97 (performance)<br>***Post-surgery:***<br>**WASI:** 118 (full scale),<br>123 (verbal), 108 (performance)	**Grooved pegboard:**<br>50th percentile<br>(dominant hand)	not done	**Working memory (from WISC-V):**<br>***Post-surgery:***<br>34th percentile	**WJ III ACH:**<br>Reading: 63rd percentile<br>Letter-Word: 67th percentile<br>Passage: 56th percentile<br>Calculation: 91st percentile

CVLT-C: California Verbal Learning Test–Children's Version
D-KEFS: The Delis–Kaplan Executive Function System
Grooved Pegboard: Grooved Pegboard for Manipulation and Dexterity Testing
WASI: Wechsler Abbreviated Scale of Intelligence
WISC-V: Wechsler Intelligence Scale for Children–Fifth Edition
WJ III ACH: The Woodcock-Johnson III Tests of Achievement
WRAML-2: Wide Range Assessment of Memory and Learning–Second Edition

Supplementary Note C. Silence localization based on the structural segmentation of MRI using *AFNI* software

As is explained in “Results”, we used the open source *AFNI* software [5, 6] to explore the effect of error in the structural segmentation of MRI on the silence localization. *AFNI* takes into account which hemisphere is intact in the brain, and along with a brain atlas (MNI152_T1_2009c+tlrc was used in our analysis [6]), it strips the skull and segments the MRI scan. Following the steps in “Methods: Data analysis”, the ground truth regions of silence are extracted from the processed MRI scans using *AFNI*. We conducted the silence localization for the three pediatric patients in our study using SilenceMap with baseline, for both the rest and the visual stimulation condition, based on the ground truth regions of silence extracted using *AFNI*. The results are included in Supplementary Fig. 3. Based on these results, there is a slight change (small reduction) in the silence localization performance (5mm, 3mm, and 7mm higher distance error (ΔCOM) for UD, SN, and OT respectively) using the *AFNI* software in comparison with the results using the *FreeSurfer* software (see Fig. 3 in our paper). This confirms that the reported pipeline for segmentation of the MRI scans in our paper using the *FreeSurfer* software is performing reasonably well and is not a significant source of error in silence localization using the scalp EEG.

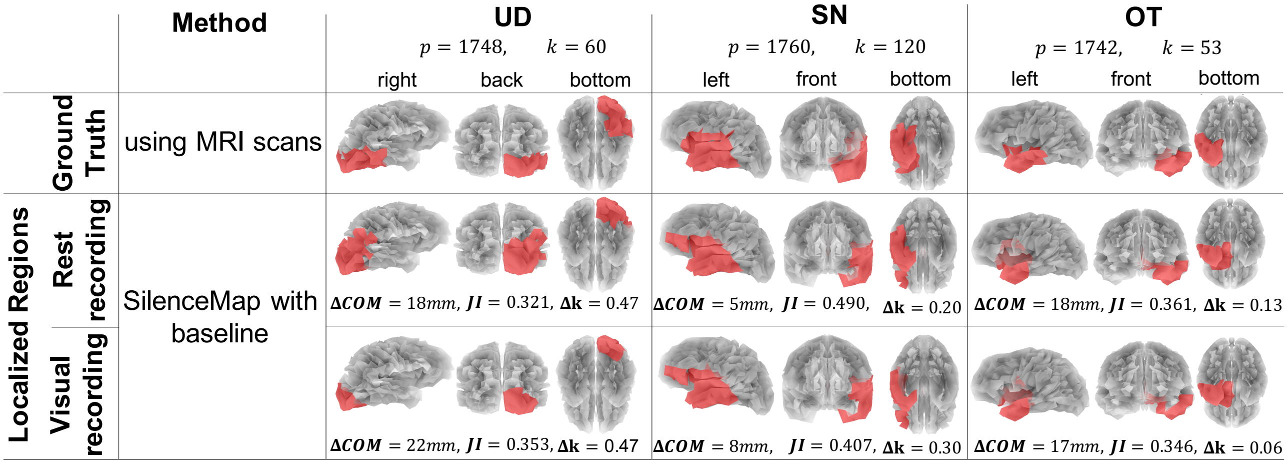

Supplementary Fig. 3. Performance of SilenceMap with baseline, for both the rest and the visual stimulation condition, based on the ground truth regions of silence extracted using the open source *AFNI* software: the first row shows the extracted ground truth regions of silence (red regions) overlaid on the resected cortical region of three patients based on their symmetric brain models extracted from the structural MRIs (see the MRI scans in Fig. 2); the second and third rows show the performance in localization of SilenceMap with baseline, based on the Rest and Visual recordings respectively, through both visual illustration (red regions) and using performance metrics of center-of-mass (COM) distance (ΔCOM), Jaccard Index (JI), and size error (Δk). p is the total number of sources in each brain model, and k is the size of ground truth region of silence. There is a slight reduction in the silence localization performance using the *AFNI* software in comparison with the results using the *FreeSurfer* software (see Fig. 3 in our paper)

Supplementary Note D. The effect of brain-to-skull conductivity ratio on the localization of the silence

Regarding the assumptions on the brain-to-skull conductivity ratio (BSCR), there is no consensus in the literature [7]. The BSCR was first estimated to be 80 in [8, 9]. This BSCR was widely accepted and used by researchers. However, in the past twenty years the value of BSCR has been the subject of much debate. In [10], Oostendorp et al. estimated a BSCR of 15 using both in vivo and in vitro experiments. In later work, Lai et al. [11] used a spherical head model to estimate the human BSCR as 24.8 from simultaneously recorded intra- and extracranial potentials in 5 epilepsy patients. However, in 2006, Zhang et al. [12] further suggested the BSCR to be 18.7 by using simultaneous intra- and extracranial recordings in two epilepsy patients. To address this comment and to further explore the effect of BSCR on the silence localization performance, we conducted the silence localization for the three pediatric patients in our study using SilenceMap with baseline, for both the rest and the visual stimulation condition, with the forward matrices (**A** in equation (5) in our paper) calculated based on the largest, and widely used BSCR of 80. The results are included in Supplementary Fig. 4. We compared the results with the silence localization

performances reported in Fig. 3 of our paper, where we have used a BSCR of 15 (the smallest BSCR reported in the literature supported by in vitro, as well as in vivo experiments [10]). Based on the results, there is only a small difference between the performance of the SilenceMap with baseline using BSCR of 15 vs. 80, in both the visual and rest recordings, which confirms the robustness of our algorithm to the potential error in the estimated forward matrix due to the assumptions on the conductivity ratios.

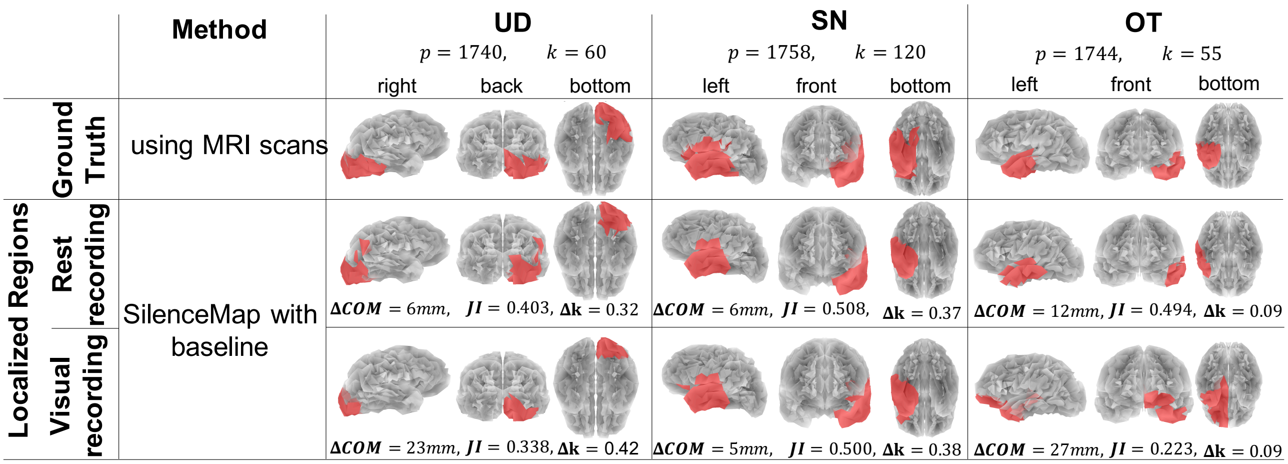

Supplementary Fig. 4. **Performance of SilenceMap with baseline, for both the rest and the visual stimulation condition, based on a large brain-to-skull conductivity ratio (BSCR) of 80:** the first row shows the extracted ground truth regions of silence (red regions) overlaid on the resected cortical region of three patients based on their symmetric brain models extracted from the structural MRIs (see the MRI scans in Fig. 2); the second and third rows show the performance in localization of SilenceMap with baseline, based on the Rest and Visual recordings respectively, through both visual illustration (red regions) and using performance metrics of center-of-mass (COM) distance (ΔCOM), Jaccard Index (JI), and size error (Δk). p is the total number of sources in each brain model, and k is the size of ground truth region of silence. The results show only small difference in the silence localization performance using a large BSCR of 80 in comparison with the results using a small BSCR of 15 (see Fig. 3 in our paper)

Supplementary Note E. Estimation of noise covariance matrix $\mathbf{C}_z$

As mentioned in "Introduction", the main difference between source localization and silence localization is in the noise definition. Most of the source localization algorithms group together the measurement noise with the background brain activity and provide methods for estimation of noise [13, 14]. However, the background brain activity is crucial for the silence localization to distinguish between normal brain activity and abnormal silence. Therefore we need to revise the noise definition accordingly. As mentioned in "Methods: Problem statement", noise is white and bandpass filtered in a specific frequency interval of $[f_L = 1, f_H = 100]Hz$ (during the preprocessing step, see "Methods: Data analysis"). f_L and f_H are the lower and the upper cutoff frequencies of the filter we have used in the preprocessing step to bandpass filter the scalp EEG signals. In addition, we assume that the noise components are spatially uncorrelated, and the noise covariance matrix is a diagonal matrix, as is defined in equation (8) in our paper. Therefore, under stationary assumption for $\widetilde{\boldsymbol{\varepsilon}}_t$, we can estimate $\widehat{\mathbf{C}_z}$ as follows:

$$(\widehat{\mathbf{C}_z})_{ii} = \widehat{\sigma}^2_{z_i} = E[\widetilde{\varepsilon}^2_{it}] = R_{\widetilde{\varepsilon}_i\widetilde{\varepsilon}_i}(0) = \frac{1}{2\pi}\int_{passband} U_{i_{white}}(j\omega)d\omega, \quad \text{for } i = \{1, 2, \cdots n-1\}, \tag{S 1}$$

where $U_{i_{white}}(j\omega)$ is the constant power spectral density of the white noise at the scalp electrode i in the frequency bands of $[f_L, f_H]$ and $[-f_H, -f_L]$, and zero outside these frequency intervals, and $\widetilde{\varepsilon}_{it}$ is the element of $\widetilde{\boldsymbol{E}}$ at t^{th} time point and i^{th} electrode (see equation (7) in our paper). This constant power spectral density can be estimated from the power spectral density of the recorded signal $y_{i,t}$ in the high frequencies ($\geq f_H - \Delta f$), where we assume that EEG does not have any frequency component and the noise power dominates:

$$\widehat{U}_{i_{white}}(j\omega) = \frac{1}{2\pi(\Delta f)}\int_{2\pi(f_H-\Delta f)}^{2\pi(f_H)} U_{y_iy_i}(j\omega)d\omega, \tag{S 2}$$

where Δf is the frequency bandwidth in which the white noise is assumed to have the dominant energy (see Supplementary Fig. 5).

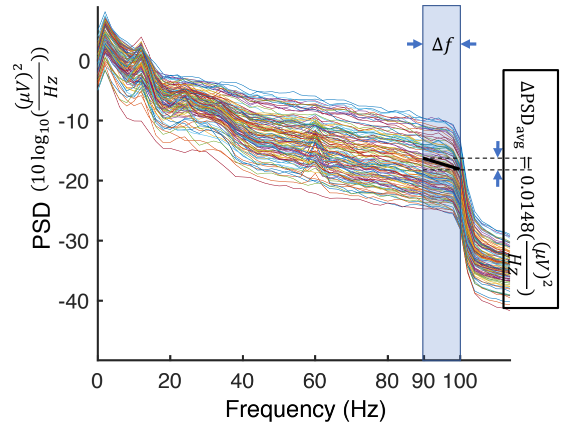

Supplementary Fig. 5. Power spectral density (PSD) of differential signals in a healthy participant (DH): The signals are bandpassed in the frequency interval of $[f_L = 1, f_H = 100]Hz$. The highlighted region is the frequency interval, during which the approximately white noise is assumed to have the dominant energy. On average, the PSD of EEG signals drops only by $\Delta PSD = 0.0148(\frac{(\mu V)^2}{Hz})$ from $90Hz$ to $100Hz$.

where $U_{y_i y_i}(j\omega)$ can be estimated from the differential signal in the ith row of $\mathbf{Y}$ (see Supplementary Note G). Modeling and estimation of EEG noise based on the PSD of the recorded signals is commonly used in EEG source localization studies [15–17], which we have modified for the silence localization task. Supplementary Fig. 5 shows the PSD of the differential signals in a healthy participant (DH), which are bandpassed in the frequency interval of $[f_L = 1, f_H = 100]Hz$ during the preprocessing step. The frequency interval, during which white noise is assumed to have the dominant energy is highlighted in this figure ($\Delta f = 10Hz$). This assumption is approximately true, i.e., averaged over $i = 1, 2, \cdots n-1$, the PSD of y_i drops only by $\Delta PSD = 0.0148(\frac{(\mu V)^2}{Hz})$ from $f_H - \Delta f = 90Hz$ to $f_H = 100Hz$ (see Supplementary Fig. 5).

Supplementary Note F. Estimation of sample variance of μ_{qt}

To estimate $Var(\mu_{qt})$ in equation (13) in our paper, average of the sample variances of μ_{qt} cannot be used since based on the WSS assumptions in "Methods: Problem statement", the elements of $\mathbf{S}$ and $\widetilde{\mathbf{E}}$, and consequently μ_{qt} are correlated over time. However, since μ_{qt} in equation (12) in our paper is a linear combination of s_{it} and $\widetilde{\mathbf{a}}_q^T \widetilde{\boldsymbol{\varepsilon}}_t$, which are both WSS and are not correlated with each other, μ_{qt} is also WSS and its variance can be estimated numerically using the time samples, as follows [18]:

$$\widehat{Var}(\mu_{qt}) = E[\mu_{qt}^2] = R_{\mu_q \mu_q}(0) = \frac{1}{2\pi}\int_{-\infty}^{+\infty} \widehat{U}_{\mu_q \mu_q}(j\omega)d\omega,, \tag{S 3}$$

where $\widehat{U}_{\mu_q \mu_q}$ is an estimation of power spectral density (PSD) of correlation coefficient μ_{qt}. We have used Welch's method to obtain this estimate [19–21], which is implemented in Matlab. A window size of 500ms, with 250ms overlap used in estimation of the PSD.

Supplementary Note G. Estimation of the variance of differential signals in Y

Similar to the estimation of the noise variance in Supplementary Note E, we estimate the variance of y_i (the ith differential signal in $\mathbf{Y}$) based on its PSD. An average of the sample variances of y_i cannot be used since based on the WSS assumptions in "Methods: Problem statement", the elements of $\mathbf{S}$ and $\widetilde{\mathbf{E}}$, and consequently y_i are correlated over time. However, since y_i in equation (11) in our paper is a linear combination of $\mathbf{s}_t$ and $\widetilde{\boldsymbol{\varepsilon}}_t$, which are both WSS and are not correlated with each other, y_i is also WSS and its variance can be estimated numerically using the time samples, as follows [18]:

$$\widehat{Var}(y_i) = E[y_i^2] = R_{y_i y_i}(0) = \frac{1}{2\pi}\int_{-\infty}^{+\infty} \widehat{U}_{y_i y_i}(j\omega)d\omega,, \tag{S 4}$$

where $\widehat{Var}(y_i)$ is the estimated variance, and $\widehat{U}_{y_i y_i}$ is an estimation of PSD of the differential signal y_i. We have used Welch's method to obtain this estimation [19–21].

Supplementary Note H. Data analysis and figures for "Real PSD" simulations

As explained in the step (i) of the "Real PSD" simulations in "Methods: Simulated dataset", we extract a general shape of PSD for the normal brain activities based on an open source real recorded electrocorticography (ECoG) dataset used in [22] and available in the open-source library in [23]. This general shape of PSD (Supplementary Fig. 6) results from averaging over the PSDs of the recordings from 62 ECoG electrodes placed around the frontotemporal region of an epileptic patient (patient "zt" in [23]).

Ethics statement: All patients participated in a purely voluntary manner, after providing informed written consent, under experimental protocols approved by the Institutional Review Board of the University of Washington (#12193). All patient data was anonymized according to IRB protocol, in accordance with HIPAA mandate. These data originally appeared in the manuscript "Rapid online language mapping with electrocorticography" published in Journal of Neurosurgery: Pediatrics in 2011 [22].

Data analysis: A Butterworth IIR filter is used to bandpass filter the ECoG signals in the frequency interval of $[0.1, 200]Hz$. In addition, we used the ZapLine method proposed in [24] to remove the power line noise components at 60, 120, and 180 Hz (see Supplementary Fig. 6). ZapLine removes the power line components with minimal degradation of other frequency components of the multi-channel ECoG data [24]. The PSDs of the filtered and noise removed signals are then averaged across the 62 electrodes to extract the general shape of PSD in Supplementary Fig. 6 (red curve). Following the steps of the "Real PSD Simulations" in "Methods: Simulated dataset", we design an FIR filter based on this average PSD (see Supplementary Fig. 7), apply this filter on the simulated non-silent source activities (generated initially with a flat PSD, see Supplementary Fig. 8) to obtain the "Real PSD" simulated signals for the non-silent sources in the brain (Supplementary Fig. 9 shows the PSD of these signals). Finally, the scalp signals are simulated based on these "Real PSD" signals, which have PSDs (see Supplementary Fig. 10) similar to the PSD of a real recorded EEG signal from a patient with a region of silence in the brain (see Supplementary Fig. 11, showing the PSD of Rest recordings for patient OT in this study, bandpass filtered in the [1,100] Hz interval). Please note that, in all of the presented PSD plots in this section, the magnitude of the real recorded signals are presented in μV, and the amplitude of the simulated signals are presented in an arbitrary unit $a.u.$, where in equation (9) in our paper $\sigma_s = 1$ $a.u.$ for simulations.

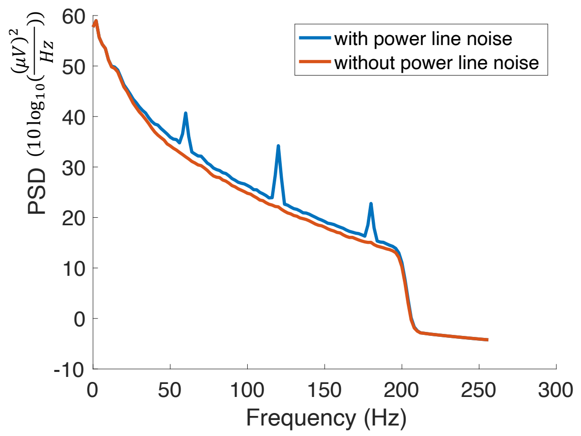

Supplementary Fig. 6. Power spectral density (PSD) of normal brain activities: Averaged PSD over 62 electrodes, based on a real recorded electrocorticography (ECoG) dataset used in [22] and available through the open-source library in [23]. The blue and the orange lines are the ECoG average PSD, with and without the power line noise components respectively (see Supplementary Note H for more details).

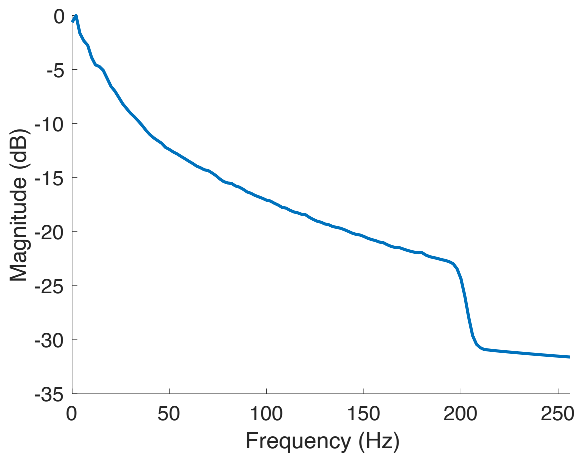

Supplementary Fig. 7. **Magnitude of a filter designed based on normal brain activity:** A linear phase finite impulse response (FIR) filter, with the magnitude of equal to the square root of the average power spectral density (PSD) shown in Supplementary Fig. 6.

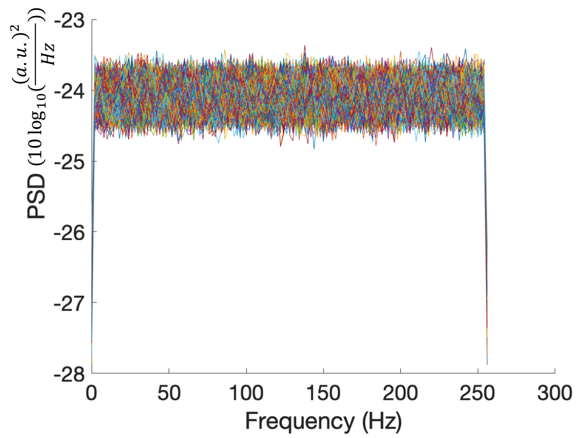

Supplementary Fig. 8. **Flat PSD brain signals simulations:** Power spectral density (PSD) of simulated non-silent source activities with independent time points.

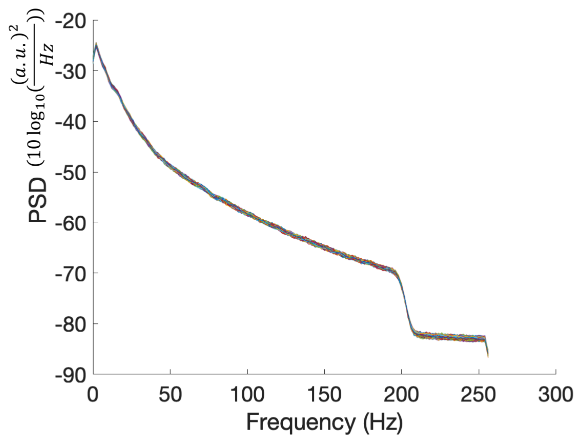

Supplementary Fig. 9. **"Real PSD" brain signals simulations:** Power spectral density (PSD) of simulated non-silent source activities based on a general shape of PSD extracted from the normal brain activities based on a real recorded electrocorticography (ECoG) dataset.

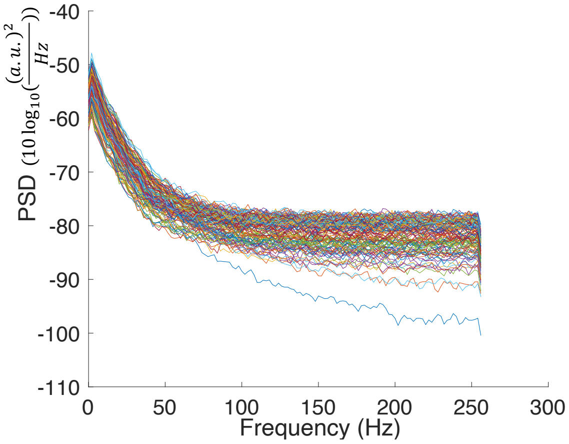

Supplementary Fig. 10. **"Real PSD" EEG simulations:** Power spectral density (PSD) of simulated scalp electroencephalography (EEG) signals based on the "Real PSD" simulated signals in the brain (see Supplementary Fig. 9).

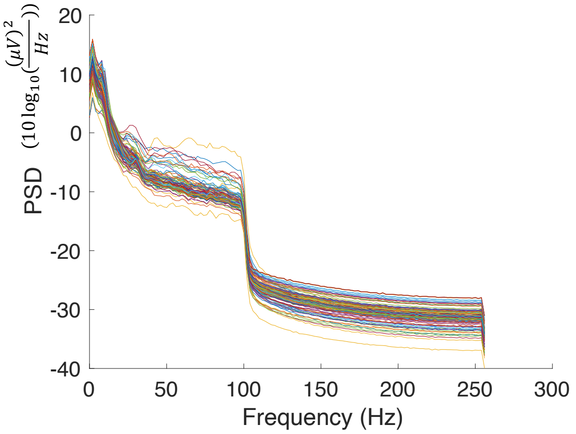

Supplementary Fig. 11. **PSD of EEG signals in a patient with resection:** Power spectral density (PSD) of differential scalp signals in a pediatric patient with resection (OT, see Table II in "Results"), during the Rest recordings. The signals are bandpassed in the frequency interval of $[1, 100]Hz$.

Supplementary References

---

[1] Alireza Chaman Zar, Marlene Behrmann, and Pulkit Grover. Pediatric patients with lobectomy (MRI and EEG). Carnegie Mellon University. Dataset, 2020. https://doi.org/10.1184/R1/12402416.v2.

[2] Alireza Chamanzar, Marlene Behrmann, and Pulkit Grover. SilenceMap: open source code and software. GitHub, 2020. https://github.com/Chamanzar/SilenceMap/tree/v1.0.

[3] Tina T Liu, Adrian Nestor, Mark D Vida, John A Pyles, Christina Patterson, Ying Yang, Fan Nils Yang, Erez Freud, and Marlene Behrmann. Successful reorganization of category-selective visual cortex following occipito-temporal lobectomy in childhood. Cell reports, 24(5):1113–1122, 2018.

[4] Tina T Liu, Erez Freud, Christina Patterson, and Marlene Behrmann. Perceptual function and category-selective neural organization in children with resections of visual cortex. Journal of Neuroscience, 39(32):6299–6314, 2019.

[5] Thorsten Joachims. Making large-scale svm learning practical. Technical report, Technical Report, 1998.

[6] Robert W Cox. Afni: software for analysis and visualization of functional magnetic resonance neuroimages. Computers and Biomedical research, 29(3):162–173, 1996.

[7] Gang Wang and Doutian Ren. Effect of brain-to-skull conductivity ratio on eeg source localization accuracy. BioMed research international, 2013, 2013.

[8] Stanley Rush and Daniel A Driscoll. Current distribution in the brain from surface electrodes. Anesthesia & Analgesia, 47(6):717–723, 1968.

[9] David Cohen and B Neil Cuffin. Demonstration of useful differences between magnetoencephalogram and electroencephalogram. Electroencephalography and clinical neurophysiology, 56(1):38–51, 1983.

[10] Thom F Oostendorp, Jean Delbeke, and Dick F Stegeman. The conductivity of the human skull: results of in vivo and in vitro measurements. IEEE transactions on biomedical engineering, 47(11):1487–1492, 2000.

[11] Y Lai, W Van Drongelen, L Ding, KE Hecox, VL Towle, DM Frim, and Bin He. Estimation of in vivo human brain-to-skull conductivity ratio from simultaneous extra-and intra-cranial electrical potential recordings. Clinical neurophysiology, 116(2):456–465, 2005.

[12] Yingchun Zhang, Wim Van Drongelen, and Bin He. Estimation of in vivo brain-to-skull conductivity ratio in humans. Applied physics letters, 89(22):223903, 2006.

[13] Christophe Phillips, Michael D Rugg, and Karl J Friston. Systematic regularization of linear inverse solutions of the eeg source localization problem. NeuroImage, 17(1):287–301, 2002.

[14] Hilde M Huizenga, Jan C De Munck, Lourens J Waldorp, and Raoul PPP Grasman. Spatiotemporal eeg/meg source analysis based on a parametric noise covariance model. IEEE Transactions on Biomedical Engineering, 49(6):533–539, 2002.

[15] Alan Paris, George K Atia, Azadeh Vosoughi, and Stephen A Berman. A new statistical model of electroencephalogram noise spectra for real-time brain–computer interfaces. IEEE Transactions on Biomedical Engineering, 64(8):1688–1700, 2016.

[16] Kenneth D Miller and Todd W Troyer. Neural noise can explain expansive, power-law nonlinearities in neural response functions. Journal of neurophysiology, 87(2):653–659, 2002.

[17] Klaus Linkenkaer-Hansen, Vadim V Nikouline, J Matias Palva, and Risto J Ilmoniemi. Long-range temporal correlations and scaling behavior in human brain oscillations. Journal of Neuroscience, 21(4):1370–1377, 2001.

[18] Alan V Oppenheim and George C Verghese. Signals, Systems and Inference: Class Notes for 6.011, Introduction to Communication, Control and Signal Processing. 2010.

[19] Peter Welch. The use of fast fourier transform for the estimation of power spectra: a method based on time averaging over short, modified periodograms. IEEE Transactions on audio and electroacoustics, 15(2):70–73, 1967.

[20] Petre Stoica, Randolph L Moses, et al. Spectral analysis of signals. 2005.

[21] Monson H Hayes. Statistical digital signal processing and modeling. John Wiley & Sons, 2009.

[22] Kai J Miller, Taylor J Abel, Adam O Hebb, and Jeffrey G Ojemann. Rapid online language mapping with electrocorticography. Journal of Neurosurgery: Pediatrics, 7(5):482–490, 2011.

[23] Kai J Miller. A library of human electrocorticographic data and analyses. Nature human behaviour, 3(11):1225–1235, 2019.

[24] Alain de Cheveigné. Zapline: A simple and effective method to remove power line artifacts. NeuroImage, 207:116356, 2020.
