# Supplementary material for: Neural silences can be localized rapidly using noninvasive scalp EEG
Source: Commun Biol. 2021 Mar 30;4:429. doi: 10.1038/s42003-021-01768-0 (PMC8010113; doi:10.1038/s42003-021-01768-0)
Supplement: Supplementary file 3 — Reporting Summary [file 42003_2021_1768_MOESM3_ESM.pdf]

nature research

Corresponding author(s): Alireza Chamanzar

Last updated by author(s): Jan 15, 2021

Reporting Summary

Nature Research wishes to improve the reproducibility of the work that we publish. This form provides structure for consistency and transparency in reporting. For further information on Nature Research policies, see Authors & Referees and the Editorial Policy Checklist .

Statistics

For all statistical analyses, confirm that the following items are present in the figure legend, table legend, main text, or Methods section.

n/a	Confirmed	
☐	☒	The exact sample size (*n*) for each experimental group/condition, given as a discrete number and unit of measurement
☐	☒	A statement on whether measurements were taken from distinct samples or whether the same sample was measured repeatedly
☒	☐	The statistical test(s) used AND whether they are one- or two-sided<br>*Only common tests should be described solely by name; describe more complex techniques in the Methods section.*
☐	☒	A description of all covariates tested
☐	☒	A description of any assumptions or corrections, such as tests of normality and adjustment for multiple comparisons
☐	☒	A full description of the statistical parameters including central tendency (e.g. means) or other basic estimates (e.g. regression coefficient) AND variation (e.g. standard deviation) or associated estimates of uncertainty (e.g. confidence intervals)
☒	☐	For null hypothesis testing, the test statistic (e.g. *F*, *t*, *r*) with confidence intervals, effect sizes, degrees of freedom and *P* value noted<br>*Give P values as exact values whenever suitable.*
☒	☐	For Bayesian analysis, information on the choice of priors and Markov chain Monte Carlo settings
☒	☐	For hierarchical and complex designs, identification of the appropriate level for tests and full reporting of outcomes
☒	☐	Estimates of effect sizes (e.g. Cohen's *d*, Pearson's *r*), indicating how they were calculated

Our web collection on statistics for biologists contains articles on many of the points above.

Software and code

Policy information about availability of computer code

Data collection	ActiView Biosemi software was used for EEG recording.
Data analysis	We used the FreeSurfer (v6.0.0), MNE (v0.14.0), and AFNI (v20.2.08) open-source software for processing the MRI scans in this study. We preprocessed the recorded EEG signals using EEGLAB toolbox (v2019.0) in MATLAB. We calculated the lead-field/forward matrix using the FieldTrip MATLAB toolbox (fieldtrip-20170828). The SilenceMap algorithm was developed in MATLAB (R2018b), using standard toolboxes, and the CVX MATLAB package (v2.1). All MATLAB code is made available online on GitHub (DOI: 10.5281/zenodo.3892185).

For manuscripts utilizing custom algorithms or software that are central to the research but not yet described in published literature, software must be made available to editors/reviewers. We strongly encourage code deposition in a community repository (e.g. GitHub). See the Nature Research guidelines for submitting code & software for further information.

Data

Policy information about availability of data

All manuscripts must include a data availability statement. This statement should provide the following information, where applicable:

- Accession codes, unique identifiers, or web links for publicly available datasets
- A list of figures that have associated raw data
- A description of any restrictions on data availability

The anonymized raw EEG dataset and MRI scans (shown in Fig.2) of the participants in this research are made available online on KiltHub, Carnegie Mellon University's online data repository (DOI: 10.1184/R1/12402416).

Field-specific reporting

Please select the one below that is the best fit for your research. If you are not sure, read the appropriate sections before making your selection.

- [x] Life sciences
- [ ] Behavioural & social sciences
- [ ] Ecological, evolutionary & environmental sciences

For a reference copy of the document with all sections, see nature.com/documents/nr-reporting-summary-flat.pdf

Life sciences study design

All studies must disclose on these points even when the disclosure is negative.

Sample size

We recruited three participants with different resections against whose data we could run the various analytic comparisons. A spatial resolution of 128 scalp EEG electrodes was used for each participant. We recorded 320 seconds of EEG data (160 seconds for Rest state and 160 seconds for the Visual task), with a sampling frequency of 512Hz, which results in a total number of 81,920 data points over time (for each task), which is considered to be a large enough sample for the statistical estimations in this study (i.e., mean, covariance, power spectral density (PSD), and noise). In addition to the recordings from the participants in this study, we simulated 100 regions of silence at 100 different random locations on a real brain model extracted from the MRI scans. This sample size of 100 regions of silence was large enough to keep the reported standard errors (SE) small. Last, we included a single control individual for further evaluation of the hemispheric assumptions.

Data exclusions

No participants, and/or simulated regions of silence, in this study were excluded from the analyses. We monitored the electrode-gel-scalp contact quality through the data acquisition period using the "Electrode Offsets" option in the ActiView data acquisition software, which calculates the DC potentials generated at the junction of the skin and electrolyte solution (gel) under the electrodes. Electrodes with larger than 20 mV offset were marked for removal and interpolation in the preprocessing step, and more conductive gels were added to the electrodes with high offset (see "Results: Real Data" for more details). Through the standard EEG preprocessing steps, which are stated in detail in "Method: Data analysis", artifact components, noisy trials, and noisy channels were removed and/or interpolated using the EEGLAB toolbox in MATLAB. Sources of noise and artifacts in the EEG recording include, but not limited to, eye blinks and eye movements, heart beats, muscle movements, line noise, jaw clenching, and weak skin-electrode contact due to drying gels.

Replication

The silence localization was repeated based on the Visual dataset for each participant, and the performance was compared to the silence localization based on the Rest dataset. The results remained largely the same, which verifies the reproducibility of the experimental findings. In addition, we repeated the silence localization for different temporal lengths of the EEG data, for each participant, and the results are compared in "Result".

Randomization

We simulated 100 different regions of silence, at random locations in the real brain model extracted from MRI scans.

Blinding

The investigator was not blinded to group allocation during the experiment. Blinding was not relevant because the main purpose of the study was to test the performance of the SilenceMap algorithm, i.e., the silence localization performance, and the required amount of data, in comparison to the state-of-the-art source localization methods.

Reporting for specific materials, systems and methods

We require information from authors about some types of materials, experimental systems and methods used in many studies. Here, indicate whether each material, system or method listed is relevant to your study. If you are not sure if a list item applies to your research, read the appropriate section before selecting a response.

Materials & experimental systems

n/a	Involved in the study	
x		Antibodies
x		Eukaryotic cell lines
x		Palaeontology
x		Animals and other organisms
	x	Human research participants
x		Clinical data

Methods

n/a	Involved in the study	
x		ChIP-seq
x		Flow cytometry
x		MRI-based neuroimaging

Human research participants

Policy information about studies involving human research participants

Population characteristics	Three male pediatric patients were recruited under IRB HS14-607 for this experiment. Two patients (SN 13y, and OT 19y) had resections to the left hemisphere and one (UD 12y) had a resection to the right hemisphere. In two of these patients (OT and UD), lobectomy surgery was performed to control pharmacoresistant epilepsy, and in the third patient (SN) surgery was performed for an emergent evacuation of cerebral hematoma at day one of life. More information about these patients is included in Table II, in "Results". In addition, we recruited a neurologically healthy control subject (DH, male, 25yr) under IRB HS13-666 to validate the hemispheric symmetry assumption in our study.
Recruitment	DH and OT signed a consent form, and the parents of SN and UD consented to their participation as they were minors. They both provided assent. All participants were compensated for their participation in the EEG recording sessions. The MRI scans were collected from the dataset used in [Maallo, et al., NeuroImage'20: DOI: 10.1184/R1/9856205]. All patients have participated in studies conducted by Marlene Behrmann under the auspices of the acknowledged NEI grant.
Ethics oversight	All procedures were approved by the Carnegie Mellon University Institutional Review Board (IRB HS13-666 for the controls, and IRB HS14-607 for the patients, "Visual recovery after severe brain injury or visual pathway disturbance").

Note that full information on the approval of the study protocol must also be provided in the manuscript.
